# Morphological Description of the Immatures of the Ant, *Monomorium floricola*

**DOI:** 10.1673/031.010.1501

**Published:** 2010-03-14

**Authors:** Daniel Russ Solis, Eduardo Gonçalves Paterson Fox, Luciane Mayumi Kato, Carlos Massuretti de jesus, Antonio Teruyoshi Yabuki, Ana Eugênia de Carvalho Campos, Odair Correa Bueno

**Affiliations:** ^1^ Centro de Estudos de lnsetos Sociais, São Paulo State University (UNESP) 13506-900, Rio Claro, SP, Brazil.; ^2^ Unidade Laboratorial de Referência em Pragas Urbanas, Instituto Biológico, 04014-002, São Paulo, SP, Brazil.

**Keywords:** larval instars, Myrmicinae, Solenopsidini, tramp species

## Abstract

Some ant species of the genus *Monomorium* Mayr occur worldwide and are considered important urban pests. The larvae of only a few species of this genus have been described, and these descriptions are either superficial or incomplete. This study aimed to determine the number of larval instars and describe the immature stages of the ant *Monomorium floricola* Jerdon (Formicidae: Myrmicinae). Specimens were analyzed and measured using light and scanning electron microscopy. Three larval instars were found, and all larvae had pheidoloid bodies with ectatommoid mandibles, consistent with other *Monomorium* species described previously. Five types of body hairs were described, and their distribution was instar-specific. Body and mandible dimensions of the larvae also were constant for each instar. Like other Myrmicinae, the larvae did not create a cocoon. Some of differences among the hair types and sensilla were observed by comparing the samples with larvae of other species in the genus, and these differences may have taxonomic utility.

## Introduction

*Monomorium* Mayr is a cosmopolitan ant genus that includes some 586 described species and subspecies (Bolton et al. 2006), some of which were spread worldwide by commerce and are now regarded as urban pests (Bolton 1987). The most important species include *Monomorium destructor* Jerdon, *Monomorium latinode* Mayr, *Monomorium floricola* Jerdon, *Monomorium pharaonis* L., *Monomorium subopacum* Smith and *Monomorium talpa* Emery (Bolton 1987). *Monomorium floricola* (Formicidae: Myrmicinae) and *M. pharaonis* are considered of even greater importance as they may be active carriers of pathogens inside hospitals (Campos-Farinha et al. 2002).

Morphological descriptions of ant larvae can provide new characters that can be useful taxonomic characters, especially for particularly difficult groups of species (Wheeler and Wheeler 1976; Schultz and Meier 1995; Pitts 2005). Moreover, improved morphological knowledge about immature stages of ants may help clarify various aspects of their biology and social organization (Fox et al. 2007).

Most ant larval descriptions were done by George C. and Jeanette Wheeler in a long series of publications describing the larvae of nearly 800 ant species (Wheeler and Wheeler 1988). However, these descriptions were usually based on few specimens and some were made without knowledge of what larval instar was being described. The latter deficiency is a consequence of the difficulties of accurately determining the number of larval instars for most insects. Of more than 11,000 extant ant species (Bolton et al. 2006) the number of larval instars is only known for 64 species from nine subfamilies, and this number typically varies between three and five instars. An updated list of these publications is presented in Table 1.

The number of *Monomorium* species that had their larvae described (Wheeler and Wheeler 1955, 1960, 1973, 1977; Petralia and Vinson 1980; Berndt and Kremer 1986; Wheeler and Wheeler 1989, 1991) was only 2% of the total number of species in the genus. The description of larvae *M. floricola* was made originally by Wheeler and Wheeler (1955); however, apart from the limitations mentioned above, it lacks important details such as body measurements.

Given the importance of larvae in ant colonies, the fact that there is no information about the number of larval instars for nearly all known ant species is a concern. Only in the last few years have some authors devoted studies to filling in this gap. Such basic knowledge is crucial to understanding immature development inside ant colonies and thus colony cycles and internal dynamics.

This investigation was aimed at determining the number of larval instars of *M. floricola* workers by measuring the growth rate between each instar, and making a detailed description of every immature stage by light and scanning electron microscopic observations.

## Materials and Methods

### Obtention of samples

Nests of *M. floricola* were obtained in 2000 in the municipalities of Piracicaba (22°43′40.03″S and 47°38′20.64″W) and Rio Claro (22°24′25.88″S and 47°33′31.37″W) in São Paulo, Brazil, and later reared in the laboratory (temperature 25±2°C and 60±10% relative humidity). From these colonies, immatures were collected for this study.

**Table 1:**
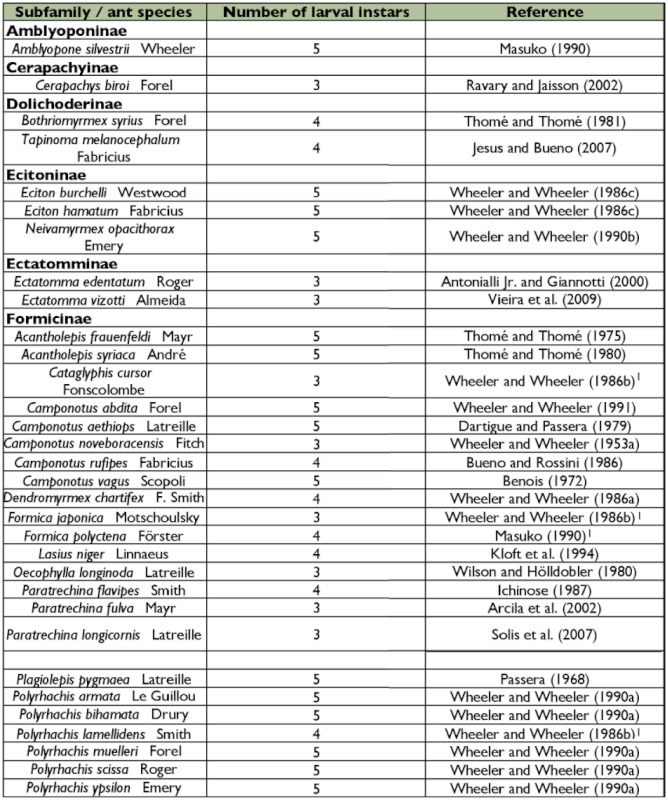
Ant species that had the number of worker larval instars determined and the respective authors. Table adapted and updated from Solis et al. (2007).

**cont.**
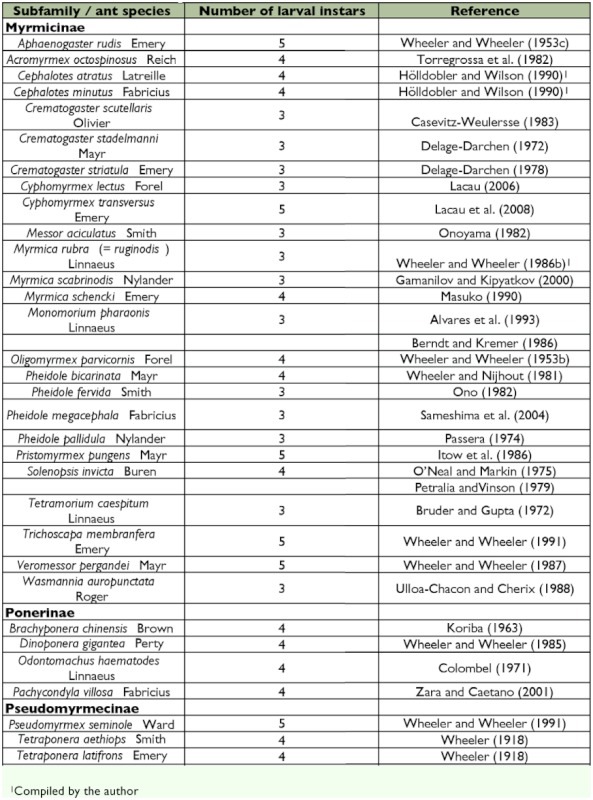

The present work focused on describing worker larvae only, as reproductive larvae can be very similar to worker larvae and thus difficult to distinguish. Knowing the general morphology of worker larvae will help identify reproductive larvae. Reproductives are usually produced during intermittent periods in much lower numbers. Therefore, care was taken to collect larvae only during periods when no reproductive forms were being produced. Workers of this species are monomorphic.

Voucher specimens of eggs, larvae, and pupae were deposited in the “Adolph Hempel” entomological collection of Centro de Pesquisa e Desenvolvimento de Sanidade Vegetal of Instituto Biológico, São Paulo, Brazil. All terminology used in our larval descriptions follows Wheeler and Wheeler (1976).

### Determining the number of larval instars

As we were unable to directly observe moults, the number of larval instars was determined using the method described in Parra and Haddad (1989). The maximum head widths of the larvae were measured (*n* = 344) and plotted in a frequency distribution graph, wherein every distinct peak was considered to correspond to a different larval instar; the obtained number of larval instars was then tested against Dyar's rule (Parra and Haddad 1989). The first larval instar and the last larval instar can be explicitly identified and used as reference to bracket others. First instar larvae are equivalent to the mature embryo, which can be measured in the egg through the transparent chorion, and last instar larvae have the developing pupa showing from within (also termed ‘prepupae’).

### Description of the immature forms

The morphological observations were made with light microscope (Zeiss MC80 DX, with maximum magnification of 1000X, www.zeiss.com) and a scanning electron microscope (Phillips SEM-505, at 12.0 kV), using ten larvae of each instar for each method. The ten larvae were selected among the ones presenting the most frequent head width found for the respective instar. With a stereomicroscope (Zeiss Stemi SV11, with maximum magnification of 66X) equipped with a micrometric eyepiece, the length and width of eggs (*n* = 159) and larvae (*n* = 50 for each instar), and length of pupae (*n* = 50) were rapidly measured. Also, body length between spiracles, a mode of measuring larvae devised by Wheeler and Wheeler (1976) that accounts for body curvature, was determined for 10 larvae of each instar. All measures are given as mean ± standard deviation, where applicable.

All collected samples were fixed in Dietrich's solution (900 ml distilled water, 450 ml 95% ethanol, 150 ml 40% formaldehyde, 30 ml acetic acid) for 24h. The samples were then transferred and conserved in 80% alcohol. For scanning electron microscope analysis, the samples were dehydrated in an acetone graded series (70–100%; specimens dipped for five min in each concentration), and critical-point dried (Balzers CPD/030, www.balzers.com). Dried specimens were then attached to aluminium stubs with double-faced conductive, adhesive tape and were goldsputtered with a Balzers SCD/050 sputterer. Observations and images were obtained as soon as possible from sample preparation. Prior to analysis under the light microscope, the larvae were warmed for 10 min in KOH 10%) and placed in a small drop of glycerine on a microscope slide.

## Results

### Determination of number of larval instars

**Figure 1:**
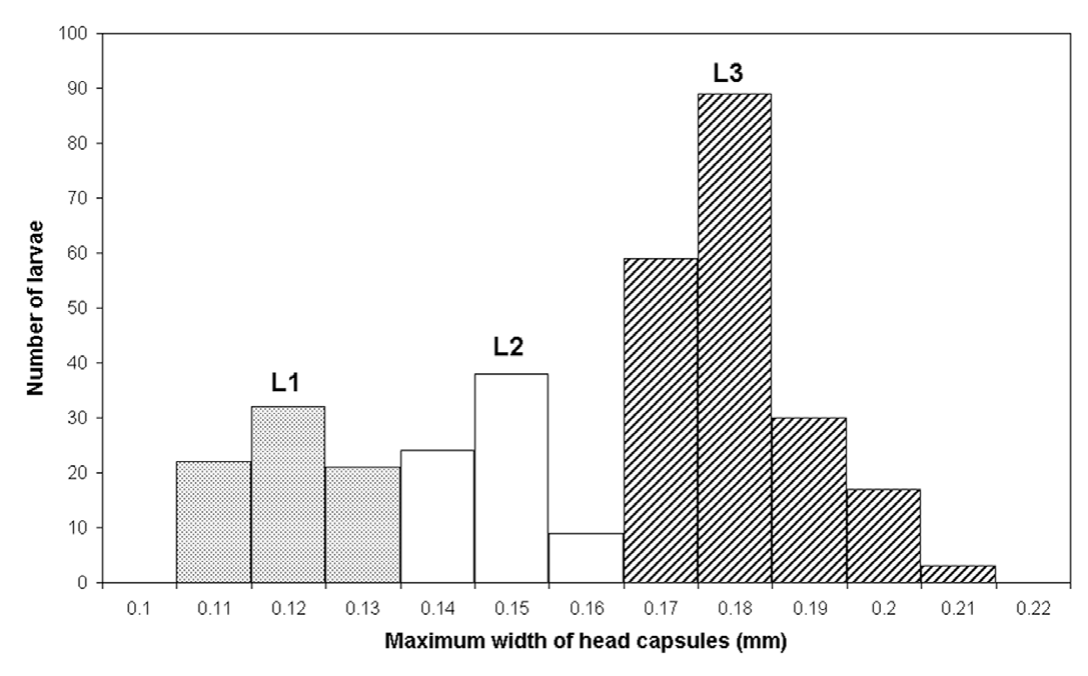
Frequency distribution of the maximum widths of head capsules of *Monomorium floricola* worker larvae: (L1) first larval instar, (L2) second larval instar, and (L3) third larval instar. The dotted region on the graph represents the interval in which mature embryos in the eggs were found and the darker traced region represents the interval in which prepupae were found. High quality figures are available online.

The frequency distribution of the maximum head widths of the larvae formed a multimodal curve with three distinct peaks (Figure 1), suggesting the existence of three larval instars. The number of instars proposed by our results yielded a good fit with Dyar's rule (R^2^ = 0.95). Mean growth rate through all instars was 1.23, while the growth rate was 1.23 between the first and second instars, and 1.22 between the second and third instars. Descriptions of the immature stages are as follows.

**Morphological description of the immature forms Egg** Ovoid in shape; chorion thin and transparent; length 0.29 ± 0.01 mm, varying 0.26–0.34 mm; width 0.18 ± 0.02 mm, varying 0.15–0.20 mm; length : width ratio was 1.61.

### First instar larva

**Figure 2:**
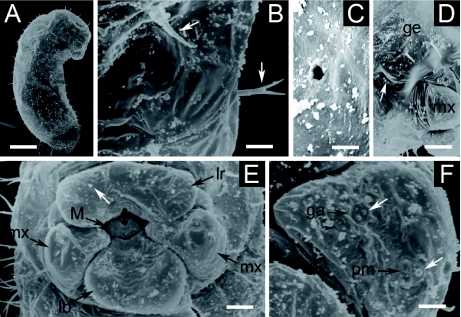
First larval instar *of Monomorium floricola*: (A) side view; (B) hair types A and B over the gena (arrows); (C) smaller abdominal spiracle; (D) close on the head capsule in side view, showing the slit-like opening (arrow): gena (ge) and maxilla (mx); (E) mouthparts: labrum (lr), maxilla (mx), mandible (M) and labium (lb), and setaceous sensilla (white arrow); (F) galea (ga) and maxillary palpus (pm), and basiconic sensilla (white arrows). Respective scale bars: 0.070 mm, 0.005 mm, 0.002 mm, 0.016 mm, 0.013 mm, 0.004 mm. High quality figures are available online.

BODY: Whitish, slender, anterior somites clearly visible, anus subterminal (Figure 2a); profile ‘pheidoloid’ as defined by Wheeler and Wheeler (1976): “Abdomen short, stout and straight; head ventral near anterior end, mounted on short stout neck, which is the prothorax; ends rounded, one end more so than the other.”

Body hairs (varying 300–400 in number; *n* = 5) uniformly distributed and organised in rows following body segmentation: most are type A hairs (0.013 ± 0.001 mm long; 0.012 – 0.014 mm; *n* = 13) and others are type B hairs (0.010 – 0.015 mm long; *n* = 6) (Figure 2b) (Table 2). Body length 0.38 ± 0.05 mm, varying 0.28 – 0.51 mm; body width 0.15 ± 0.01 mm varying 0.12 – 0.18 mm. Ten pairs of unornamented spiracles (Figure 2c) measuring 0.003 ± 0.001 mm and varying 0.002 – 0.008 mm (*n* = 100); first thoracic pair larger than the others, which are all of roughly the same size. Body length through spiracles 0.57 ± 0.17 mm, varying 0.33 – 0.76 mm (*n* = 10).

HEAD CAPSULE: Cranium 0.12 mm wide (*n* = 32); subcircular, with a slit-like opening just under the border between the gena and the first thoracic segment (Figure 2d). Twenty-six head hairs: 4 along the ventral border of the clypeus; 6 hairs on each gena; 4 on frons; 2 on vertex; and 4 along the occipital border. In almost all larvae, those were type A hairs (0.011 ± 0.002 mm long; 0.009 – 0.014 mm; *n* = 10), but in some specimens there was a type B hair (0.010 mm long) on the gena, instead of a type A hair. Well defined clypeus with no sensilla.

**Table 2:**
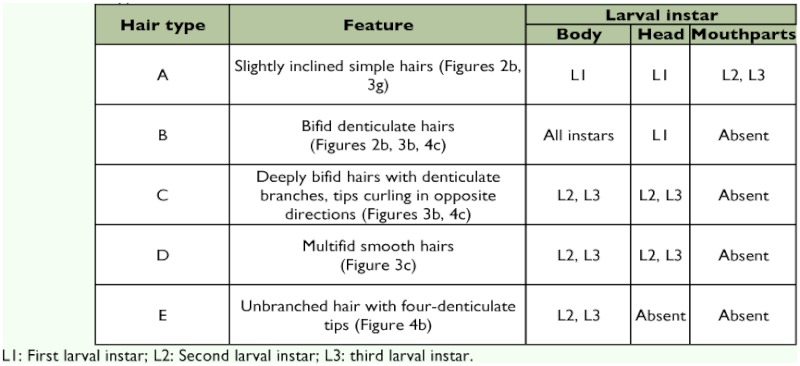
Hair types of *Monomorium floricola* larvae.

**Figure 3:**
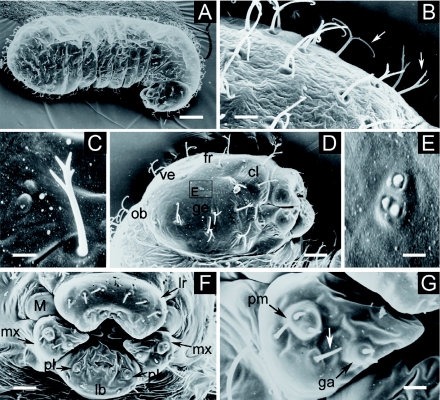
Second larval instar of *Monomorium floricola*: (A) side view; (B) hair types B and C around the anus (arrows); (C) hair type D on the gena; (D) head capsule in side view: gena (ge), clypeus (cl), frons (fr), vertex (ve) and occipital border (ob); (E) antenna with three basiconic sensilla (close on demarked area in Figure 3D); (F) mouthparts: labrum (lr), maxilla (mx), mandible (M), labium (lb) and labial palpus (pl); (G) maxilla with galea (ga) and maxillary palpus (pm), and hair type A (white arrow). Respective scale bars: 0.083 mm, 0.010 mm, 0.004 mm, 0.026 mm, 0.003 mm, 0.013 mm, 0.005 mm. High quality figures are available online.

MOUTHPARTS (Figure 2e): Labrum 0.065 mm long and 0.023 mm wide; bilobed and with 6 setaceous sensilla (0.002 mm long; *n* = 3) over the anterior surface. Mandibles 0.040 ± 0.003 mm long, varying 0.035 – 0.045 mm (*n* = 10); profile ‘ectatommoid’ as defined by Wheeler and Wheeler (1976): “Subtriangular; with a medial blade arising from the anterior surface and bearing one or two medial teeth; apex curved medially to form a tooth”; weakly sclerotized and bearing 3 teeth. Maxilla conoidal (Figure 2f); 0.037 mm long and 0.030 mm wide; maxillary palpus a skewed peg with two basiconic sensilla; digitiform galea culminating with two basiconic sensilla. Elliptical labium 0.044 mm in length; surface smooth with 4 setaceous sensilla on its ventral border; labial palpus digitiform with a setaceous sensillum on the top.

### Second larval instar

**Figure 4:**
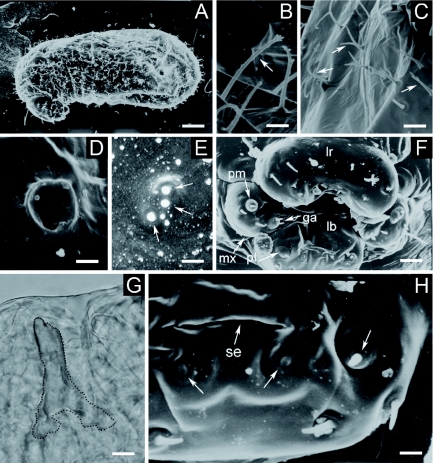
Third larval instar of *Monomorium floricola*: (A) side view; (B) hair type E on the ventral thoracic region (arrow); (C) hair types B, C, and D around the anus (arrows); (D) smaller abdominal spiracle; (E) antenna with three basiconic sensilla (arrows); (F) mouthparts: labrum (lr), maxilla (mx), galea (ga), maxillary palpus (pm), labium (lb) and labial palpus (pl); (G) mandible with three small teeth; (H) close on the labium, displaying the opening of sericteries (se) and setaceous and basiconic sensilla (arrows). Respective scale bars: 0.100 mm, 0.007 mm, 0.012 mm, 0.002 mm, 0.002 mm, 0.010 mm, 0.012 mm, 0.002 mm. High quality figures are available online.

BODY: Whitish; slender and pheidoloid in profile; distinct anterior somites; anus subterminal (Figure 3a). Body hairs (varying 400–500 in number; *n* = 5) uniformly distributed in rows following body segmentation; most were type C hairs (0.029 ± 0.002 mm long; 0.026 – 0.033 mm; *n* = 15) (Figure 3b) (Table 2). There were also hairs over the ventral region, thorax and around the anus: type D hairs (0.018 – 0.023 mm long; *n* = 5) (Figure 3c), type E hairs (0.022 – 0.028 mm long; *n* = 2) (Figure 4b), and some type B hairs (0.02 mm long) (Figure 3b) (Table 2). Body length was 0.51 ± 0.06 mm long, varying 0.38 – 0.66 mm; body width 0.18 ± 0.02 mm, varying 0.15 – 0.24 mm. Ten pairs of unornamented spiracles measuring 0.006 ± 0.002 mm and varying 0.005 – 0.013 mm (*n* = 100); first thoracic pair being larger than others, which are of similar size. Body length through spiracles was 0.77 ± 0.07 mm, varying 0.68 – 0.87 mm (*n* = 10).

HEAD CAPSULE (Figure 3d): Cranium 0.15 mm wide (*n* = 38); subcircular. Antennae with three basiconic sensilla over a slight elliptical elevation (0.006 mm wide and 0.004 mm high) (Figure 3e). Twenty-six head hairs of two types: 4 type D hairs on the ventral border of the clypeus; 6 hairs over gena, 4 of type C and 2 of type D (the latter placed near the border of the clypeus); 2 type C and 2 type D hairs over frons (the latter over the frons bordering the clypeus); 2 type C hairs over vertex; and 4 type C hairs along occipital border. Type C hairs 0.022 – 0.030 mm long (*n* = 4) and type D hairs 0.015 – 0.023 mm long (*n* = 9). Well defined clypeus with no sensilla.

MOUTHPARTS (Figure 3f): Labrum bilobed, 0.067 mm long and 0.028 mm wide and with 6 type A hairs (0.005 – 0.009 mm long; *n* = 9) over its anterior surface, 4 setaceous sensilla (0.002 – 0.003 mm long; *n* = 7) and 2 basiconic sensilla over the ventral surface, always a basiconic one in association with a setaceous one. Mandibles 0.051 ± 0.002 mm long varying 0.048 – 0.055 mm (*n* = 10), ectatommoide in shape, moderately sclerotized and bearing three teeth, with a basiconic sensillum at the base. Maxilla conoidal (Figure 3g), 0.033 mm long and 0.024 mm wide with 2 type A hairs (0.004 – 0.009 mm long; *n* = 9); in some specimens one of the hairs was replaced by a setaceous sensillum (0.003 mm long). Maxillary palpus a ‘skewed peg’ (0.01 mm long, 0.007 mm wide and 0.006 mm high) with 2 setaceous sensilla (0.002 – 0.003 mm long; *n* = 4); galea digitiform (0.004 mm long, 0.003 mm wide and 0.01 mm high) with 2 setaceous sensilla (0.002 – 0.003 mm long; *n* = 6) on the top. Labium elliptical, 0.05 mm long and devoid of spinules; digitiform labial palpus culminating with a setaceous sensillum (0.002 – 0.003 mm long; *n* = 5) and with 4 type A hairs (0.004 – 0.010 mm long; *n* = 5) on its ventral border. There was a basiconic sensillum beneath each extremity of the opening of sericteries, which is a horizontal slit.

### Third larval instar

BODY: Whitish; slender and pheidoloid in profile; distinct anterior somites; anus subterminal (Figure 4a). Body hairs (varying 500–600 in number; *n* = 5) uniformly distributed and organized in rows following body segmentation; most are type C hairs (0.028 ± 0.003 mm long; 0.024 – 0.034 mm; *n* = 20), but also type B hairs (0.014 – 0.017 mm long; *n* = 5), type D hairs (0.019 – 0.024 mm long; *n* = 5) and type E hairs (0.028 – 0.031 mm long; *n* = 3) are present and located on ventral region, thorax (Figure 4b) and around anus (Figure 4c). Body length 0.86 ± 0.19 mm, varying 0.51 – 1.26 mm; body width 0.35 ± 0.09 mm, varying 0.22 – 0.56 mm. Ten pairs of unornamented spiracles (Figure 4d) measuring 0.006 ± 0.002 mm, varying 0.005 – 0.013 mm (*n* = 100); first pair bigger than others, which were all of roughly same size. Body length through spiracles 1.16 ± 0.22 mm, varying 0.84 – 1.46 mm (*n* = 10).

HEAD CAPSULE: Cranium 0.18 mm wide (*n* = 89); subcircular. Antennae are slight elliptic 0 elevations (0.007 mm wide and 0.004 mm high) with three basiconic sensilla (Figure 4e). Twenty-six head hairs of two types: 4 type Dhairs on clypeus, along its ventral border; 4 type C hairs and 2 type D hairs are located over gena, with one of the latter located near the clypeal border; 2 type C and 2 type D hairs over frons, with the last two bordering the clypeus; 2 type C hairs on vertex; 4 type C hairs along the occipital border. Type C hairs .023 – 0.028 mm long (*n* = 8) and type D hairs 0.019 – 0.022 mm long (*n* = 4). Well-defined clypeus with no sensilla.

MOUTHPARTS (Figure 4f): Labrum bilobed about 0.068 mm long and 0.028 mm wide with 6 type A hairs (0.007 ± 0.001 mm long; 0.005–0.008 mm; *n* = 11) over the anterior surface, 4 setaceous sensilla (0.002 mm long; *n* = 4) and 2 basiconic sensilla over the ventral surface; each basiconic sensillum came in association with a setaceous sensillum. Mandibles 0.056 ± 0.005 mm long, varying 0.048 – 0.063 mm (*n* = 10); ectatommoid in shape (Figure 4g); moderately sclerotized and with three teeth, bearing a basiconic sensillum at their base. Maxilla conoidal, 0.031 mm long and 0.029 mm wide with 2 type A hairs (0.004–0.008 mm long; *n* = 4); maxillary palpus a skewed peg with 2 setaceous sensilla (0.003 mm long; *n* = 3); galea digitiform culminating with 2 setaceous sensilla (0.003 mm long; *n* = 3). Labium elliptical, 0.05 mm long; smooth surface; labial palpus digitiform with 1 setaceous sensillum on the top (0.002 mm long; *n* = 3) and 4 type A hairs (0.004– 0.005 mm long; *n* = 4); a basiconic sensillum placed on each extremity of the opening of sericteries (Figure 4h). One specimen (out of ten analyzed) had a digitiform maxillary palpus (only the right one) with a setaceous sensillum on top.

### Pupa

Exarate, with no cocoon; whitish when young, with surface and eyes getting darker on late metamorphosis. Body length 1.42 ± 0.07 mm, varying 1.26 – 1.54 mm (only white pupae were measured).

## Discussion

### Determination of number of larval instars

The number of instars herein recorded for *M. floricola* was the same as in *M. pharaonis* (Table 1). The occurrence of three larval instars is known to four ant subfamilies. This was recorded to 13 myrmicine species including *M. pharaonis* (Table 1).

The larval growth rate observed agrees with the Dyar principle, which states that the head capsule of larvae grows in geometric progression over the ecdises at a constant rate that varies between 1.1 and 1.9, usually around 1.4 (Parra and Haddad 1989).

### Morphological description of the immature forms

The original larval description of *M. floricola* by Wheeler and Wheeler (1955) was based on their description in the same paper of *M. pharaonis*. Therein the larvae were separated as ‘very young’ and ‘young’ specimens, possibly corresponding to the first and third instars judging from the stated specimen size and body hairs. Their number of analyzed specimens was specified as “numerous.” Moreover, in that description, many body measures of the larvae were not taken. In the present study, many gaps have been filled in by measuring mouthparts, head capsule, spiracles, body width and length, and the body length between spiracles.

Wheeler and Wheeler (1960) attempted to set parameters for a genus-level taxonomy of ant larvae. The main traits they considered when building an identification key to ant larvae was the body shape of the larvae in side view, the shape of mandibles and the hair types present. These traits are discussed, in this same sequence, below, while comparing them in different related ant species.

The body shape herein observed for *M. floricola* larvae agrees with observations by Wheeler and Wheeler (1976) for *Monomorium*.

From comparing the body measures of *M. floricola* larvae with those of *M. pharaonis* (Berndt and Eichler 1987), it was verified that both species have first instar larvae of approximately the same size, while second and third instar larvae of *M. pharaonis* are always larger (first instars measure 0.39 mm long and 0.18 mm wide, while the others respectively measure 0.60 mm long and 0.26 mm wide, and 1.27 mm long and 0.59 mm wide).

The mandibles of *M. floricola* larvae confirm the statement made by Wheeler and Wheeler (1976) that all hitherto described *Monomorium* larvae have ectatommoid mandibles, thus this trait remains reliable for separating larvae from different genera of Myrmicinae. Based on the observations outlined here, one can promptly sort out first instar larvae of this species as those with completely unsclerotised mandibles. This is useful because directly dissecting and measuring mandibles is very difficult.

In addition to two hair types previously recorded by Wheeler and Wheeler (1955) for *M. floricola*, three other types have been identified. Body hair distribution and types of hairs was typical of different instars (Table 2): simple body hairs were typical of first instars, while the curved bifid, multifid and unbranched denticulate hairs were typical of second and third instar larvae. Denticulate bifid hairs were found in all. All these hair types, except the unbranched denticulate ones, were also observed in larvae of *M. pharaonis* (Wheeler and Wheeler 1955; Petralia and Vinson 1980; Berndt and Eichler 1987), with the latter also having two different hair types: deeply three-branched with tips curved in opposite directions (Wheeler and Wheeler 1955) and another unbranched hook-like smooth hair (Berndt and Eichler 1987). *Monomorium antarcticum* Smith larvae had three hair types (Wheeler and Wheeler 1955), including one similar to the unbranched denticulate hairs found in *M. floricola* occurring on the thoracic area. *Monomorium afrum* André larvae were reported to bear two types of hair (Wheeler and Wheeler 1955), with one of them similar to the bifid curved hairs herein described.

All the head hairs of the mature larvae observed in this study were branched, in accordance with the Wheeler and Wheeler (1960) identification key for Myrmicinae larvae, with the exception of *M. antarcticum*. The use of head hairs differences between larvae of different species was proposed by Pitts et al. (2005) to aid species identification in the troublesome *Solenopsis saevissima* Smith complex of fire ant species. Given the proximity of *Solenopsis* to *Monomorium* (Bolton 1987), it is possible that a similar approach would be useful in separating larvae of closely related species. It should be noted that Fox et al. (2007) detected intraspecific variation in head hair types in *Paratrechina longicornis* Latreille larvae of the same instar and questioned the true applicability of such characters, while also remarking on the low number of specimens analyzed for Wheelers' descriptions. Moreover, in the present description variation in the morphology of head hairs was detected among different specimens of first instar larvae.

In comparing the labrum of mature larvae of *M. floricola* with those of the other species described in Wheeler and Wheeler (1955), it was observed they differ on the labrum from *M. pharaonis* by having between six and eight bifid hairs on the anterior surface, where mature larvae of *M. floricola* have six simple hairs. Larvae of *M. antarcticum* present between ten and twelve short hairs or sensilla on the labrum (Wheeler and Wheeler 1955).

The sensilla on the ventral surface of the labrum, as well as the sensilla near the extremities of the spinneret, base of mandibles and on the antennae, were not observable in first instar larvae, although some of them might be present. These specimens were covered in debris precluding the observation of details. The second and third instar larvae possessed hairs on the maxillae and setaceous sensilla on the maxillary palps and galea, while first instar larvae had only basiconic sensilla and no hairs on the maxillary region and parts.

Ant larvae generally have ten pairs of spiracles, two thoracic and eight abdominal; in 50%) of the genera, spiracles are relatively small and uniformly sized (Wheeler and Wheeler 1976). The first thoracic pair of spiracles of *M. floricola* was always bigger than the others, which are all uniform. The same was observed in *M. pharaonis, Monomorium tambourinensis* Forel, and *M. antarcticum* (Wheeler and Wheeler 1955; Wheeler and Wheeler 1960); this could be a recurrent trait in this genus. This was already proposed by Wheeler and Wheeler (1976), but other species must be looked at in order to confirm the pattern.

As stated by Wheeler and Wheeler (1976), all ants have silk glands, but some of them, including the Myrmicinae, do not weave cocoons. *M. floricola* fit this pattern.

Finally, this study presented a deeper analysis of the morphology of the larvae of *M. floricola*, previously described by Wheeler and Wheeler (1955), while comparing it with the few other *Monomorium* larvae described so far and confirming general similarities, e.g. the body and mandible formats. Also, the number of instars for this species was established. The different types of hair described and other findings that have direct application for instar separation with this species are particularly important. Some of the information herein may aid future systematic and taxonomic studies with the group, as well as help clarify some aspects of the biological and social organization of this ant.
